# Intrauterine growth restriction and its associated factors in South Gondar zone hospitals, Northwest Ethiopia, 2019

**DOI:** 10.1186/s13690-020-00475-2

**Published:** 2020-09-29

**Authors:** Desalegn Tesfa, Melaku Tadege, Alemayehu Digssie, Sofonyas Abebaw

**Affiliations:** Department of Public Health, College of Health Sciences, Debre Tabor University, Debretabor, Ethiopia

**Keywords:** IUGR, Associated factors, Small for gestational age, Low birth weight

## Abstract

**Background:**

After prematurity, intrauterine growth restriction (IUGR) is the second leading cause of perinatal mortality. IUGR has significant consequences in fetal, neonatal, and adult life. Currently, Ethiopia lacks information on IUGR’s prevalence and its determinants. This study aimed to assess the proportion of IUGR at birth and its associated factors.

**Methods:**

A cross-sectional study was carried out among women who give birth in four hospitals of south Gonder zone from November 2018 to February 2019. Multi-stage sampling was applied to select the required samples. IUGR was assessed using a standardized cutoff percentile/mean for each measurement. Data were collected by trained MSc clinical midwives. Bi-variable and multivariable logistic analyses were deployed to identify the association.

**Results:**

A total of 803 maternity women were participating in this study with a response rate of 95%. The proportion of IUGR 23.5% (95% CI: 20.7–26.6), low birth weight 13.3%, small-for- gestational-age 19.7%,and preterm birth 23.16%. Women who was unable to read and write, (AOR; 2.46, 95% CI: 1.02–5.92), total family size ≥7 (AOR; 1.67, 95% CI: 1.04–2.66), maternal mid-upper arm circumference (MUAC) < 23 cm (AOR; 2.10, 95% CI: 1.39–3.01), body mass index (BMI) < 18.5 kg/m^2^ (AOR; 2.57, 95% CI: 1.72–3.83), altitude > 3000 m (AOR; 1.89 95% CI: 1.19–3.01), small placental size (< 350 g) (AOR; 2.42, 95% CI: 1.67–3.54) and small-for-gestational-age (AOR; 1.94, 95% CI:1.86–4.52) were the most predictors of IUGR.

**Conclusions:**

IUGR was a major public health concern in this study. Women who were unable to read and write, small-for-gestational-age, maternal BMI < 18.5 kg/m^2^, family size ≥7, maternal MUAC < 23 cm, small placental size, and altitude > 3000 m were found the most predictor variables. Strengthen female education, nutritional intervention before and during pregnancy, and routine maternity care is critical. Further clinical follow-up research is essential which includes maternal, fetal, and placental gens.

## Background

Intrauterine growth restriction (IUGR) is defined as the velocity of fetal growth less than the normal fetus growth potential for a specific neonate or it is the failure of the fetus to achieve its growth potential [1]. In the womb life or during the postnatal period an infant with birth weight or birth length below the 10th percentile is known as small for gestational age [2, 3]. IUGR is not synonymous with small- for- gestational- age (SGA), or fetal malnutrition (FM). Because the situation may exist with or without these conditions in any newborn [4]. The identification of IUGR is commonly made during the antenatal period; however, it can be detected during the newborn period immediately after delivery [5, 6] by using clinical examination [3, 7, 8], anthropometry index [9], and clinical assessment of nutritional status (CAN) score [4]. IUGR is a public health problem and noted to affect approximately 10–15% of pregnant women.

IUGR is observed in 23.8% of the newborn and approximately 30 million babies globally suffered from it every year. Closely, 75% of all exposed newborns were occurred in developing regions [10]. The prevalence of IUGR in Malawi and Karachi was around 21% [11, 12]. Screening of neonatal adverse birth outcomes including IUGR is very important for obstetricians and perinatologists. Because its effect is associated with perinatal morbidity and mortality [13], birth hypoxia, impaired neurodevelopment, a manifestation of the metabolic syndrome in adult life [14]. It also leads to early and late complications, increases significantly in newborn birth weight, length, and head circumference less than the 10th percentile. Next to prematurity, IUGR is the second leading cause of perinatal mortality which is still the huge problem of developing countries.

IUGR fetus has approximately five to ten-fold increased risk of dying in the womb, with up to 23 to 65 stillbirths [14, 15]. Around half of the preterm stillbirths and 25% of the term, stillbirths were growth-retarded [16]. The greatest incidence of intrauterine growth restriction in developing countries is multi-factorial and involves a complex collaboration between fetal, placental, and maternal factors even though the maternal factors are the most predominant causes [10, 17]. So far, data is not available in Ethiopia which focused on this public health significant issue. This study is vital to assess the prevalence of intrauterine growth restriction and associated factors especially the maternal and placental factors in Ethiopia. Showing the proportion and associated factors of IUGR is very critical. In addition to this, early intervention could be suggested to achieve the sustainable development goal of child health. And the study is important in Ethiopia that perinatal mortality is still very high. Besides, this study finding will be used as baseline data for clinicians to do prospective clinical research.

## Methods

This study was carried out in the selected four Hospitals of South Gondar zone (three primary Hospitals which include: Nefas- Mewicha, Mekane Eyesus, and Addis Zemene), and one general Hospital (Debre Tabor). South Gondar is a Zone that belonged in the Ethiopian Amhara Regional state. Based on the 2007 Census conducted by the Central Statistical Agency of Ethiopia (CSA), this Zone has a total population of 2,051,738 and an increase of 16% over the 1994 census. In Ethiopia, even though there is a substantial increment of institutional delivery from 5% in 2005 to 48% in 2019, still greater than half (52%) of the women have been delivered at home [18]. As the 2018 South Gondar zone report, around 527,967 reproductive-age groups mother, and 87, 955 pregnant women were found in the zone. The zone has 8 Hospitals, 94 Health centers, and 378 Health posts. In each Health post, at least two Health extension workers are assigned.

The socioeconomic status of the population those attend in these four hospitals are almost similar even those the type of crop production and altitudes are different. All of the hospitals give similar services except Debre Tabor general hospital that serves as a referral center for -private and governmental health institutions. Averagely 119, 293, 97, and 108 deliveries were conducted in 2 months in Nefas- Mewicha, Debre Tabor, Mekane Eyesus, and Addis Zemene Hospitals respectively.

The study was hospital-based and cross-sectional in design. The study took place from November 2018 to February 2019.

All pregnant women who delivered in South Gondar zone Hospitals and all pregnant women who delivered in south Gondar zone selected Hospitals were the source and study population respectively.

All women who give birth in the specified period (women who deliver within four-months) and singleton live-birth were included in the study. Whereas, women who couldn’t answer the intended questions because of illness and mental problems; mothers die because of complications of labor, referred to other higher health institutions, birth with incomplete placenta, and newborn with congenital abnormality were excluded.

The sample size was determined using the formula of a single population proportion with the assumption of the prevalence of intrauterine growth restriction 50% since there is no study in Ethiopia, Z a/_2_ = 1.96 with 95 confidence interval, 5% of marginal error, and design effect 2. Then, the final sample size with a10% non-response rate was 845.

The multi-stage sampling procedure was employed to select the required sample size. Since we can’t address all the hospitals, first of all, four of the hospitals (Addis Zemen, Nefas -Mewcha, Mekane-Eyesus, and Debre Tabor) were selected randomly. Then we applied a proportional allocation for each Hospital. Based on the previous four-month delivery flow rate; we applied a systematic sampling method after determining the interval. Intrauterine growth restriction was the outcome variable in this study.

### Intrauterine growth restriction (IUGR)

Assessed using a clinical examination of 10 typical features [3, 7, 8], anthropometric index like weight-length ratio [9], fetal growth ratio which is defined as the ratio of the observed birth weight to the mean birth weight for gestational age. The infant is classified as not growth restricted if the fetal growth ratio is between 0.9–1.1, mild growth-retarded if the ratio was 0.8–0.85, moderately growth-retarded if the ratio was between 0.75–0.8, and severely growth-retarded if the ratio was below 0.75 by using curve cutoff points [19, 20], neonatal ponderal index and body mass index (BMI) was calculated as a neonatal ponderal index (NPI) = 100x [birth weight (gram) / length (cm)^3^]; BMI=Birth weight (gram)/ length (cm)^2^ [20, 21] and clinical assessments of nutritional status by using observation and a hands-on estimate of the loss of subcutaneous tissue and muscles (CAN) score [4] which is simple and rapid. We use the mean/ the cut values (percentiles) for each measurement in this study.

Small for gestational age (SGA): is defined as birth weight less than 10th centile for gestational age using weight percentile, length percentile, and head circumference percentile charts [22].

Clinical Assessment of Nutrition (CAN score): is a scoring system based on nine superficial readily detectable signs of malnutrition in the newborn baby [23].

Rapid assessment of gestational age at birth: gestational age derived from the total scores of skin texture (4 items), skin color (4 items), breast size (4items), and ear firmness (4 items) [6, 24]. But for this study gestational age was estimated by LMP (if she knows her LMP), if she didn’t know her LMP, we used ultrasound estimation during delivery, if two of them were impossible, we used a rapid assessment of gestational age estimation at birth.

Preeclampsia was defined gestational hypertension or postpartum hypertension, as defined above, developing for the first time after delivery with proteinuria (24- h urinary protein level of > 300 mg, a spot urine protein: creatinine ratio of > 300 mg/mmol creatinine, or urine dipstick protein level > 2=/1 g/l) or any multisystem complication of preeclampsia.

Household wealth status: is computed by principal component analysis from different variables such as the presence of own farmland, own toilet facility, bank account, mobile phone, electricity, the roof of the house with corrugated iron sheets, number of cows/oxen, horses/ mules/donkeys goats/sheep and chicken [25].

After reviewing the relevant literature, questionnaires were designed to include all possible variables that address the intent of this study.

Firstly, the questionnaire was developed in English and translated into the local language (Amharic). Finally, retranslated back into English to check the consistency. We prepared weight, height measuring instruments, and standardized charts/ cutoff points. For each Hospital four data collectors and 2 supervisors have participated.

To ensure the quality of data, training was given for all data collectors at each Hospital for 1 day on the over-all procedure of data collection by investigators and pediatrician supported with video. The questionnaire was pre-tested before the actual data collection time on 42 participants (5% of the sample). Weight, high, abdominal, chest and head circumference of the newborn baby was measured immediately after birth and recorded into the nearest decimals. Detailed examination of each baby carried out by data collectors.

The supervisors closely follow the day-to-day data collection process and ensure the completeness and consistency of the questionnaire administered each day. The collected data reviewed and checked for completeness before data entry.

Data clean up and cross-checking was done before analysis. Then, the collected data were checked for completeness, coded, and cleaned. This data was entered using EPI INFO -version 7 for windows statistical software. Then data were exported to SPSS version-20 for further analysis. Both descriptive and analytical statistical procedures were utilized. Only variables in binary screening had a *p*-value ≤0.2 considered in multivariable logistic regression.

Logistic regression analysis was applied to describe the functional independent predictors. Odds ratio (OR) with a 95% confidence interval (CI) was built to assess the strength of association between independent and dependent variables. For all, statistical significance was declared at p-value < 0.05.

## Results

### Socio-demographic characteristic of the respondents

Of all, 803 maternity women were participating in this study and obtaining a response rate of 95%. In this study, around two-thirds of 518 (64.5%) of the mothers were found in the age category of 20–29 years and only 308 (38.4%) of them were attending their education primary and above. Almost all 787 (98%) of the mothers were living in union with their husbands and around half 453 (56.4%) of the mothers were living with a total family size of three and less when we see the wealth status of the respondents, around half 412 (51.3%) and less than one forth 190 (23.7%) were found in the middle and better wealth quintile respectively. During their pregnancy time, 49 (6.1%) of the women have taken different medications (Table 1).

**Table 1:** Socio-Demographic Characteristics of the Respondents in South Gondar Zone Hospitals, Northcentral Ethiopia, 2019

Characteristics	Frequency	Percent
**Current maternal age (year)**		
≤ 19	56	7.0
20–29	518	64.5
30–39	203	25.3
≥ 40	26	3.2
M**aternal age during 1st pregnancy**		
< 20	508	63.3
≥ 20	295	36.7
**Educational status of the mother**		
Unable to read and write	222	27.6
Able to read and write	273	34.0
Primary	81	10.1
Secondary	113	14.1
College and above	114	14.2
**Maternal occupation**		
Housewife	496	61.8
Farmer	97	12.1
Daily worker	51	6.4
Merchant	159	19.8
**Marital status**		
Married	787	98
Single	6	0.7
Divorced	10	1.2
**Age of the husband /friend**		
< 20	12	1.5
20–29	301	37.5
30–39	354	44.1
≥ 40	136	16.9
**Husband/friend educational status**		
unable to read and write	186	23.2
able to read and write	215	26.8
Primary	66	8.2
Secondary	195	24.3
college and above	141	17.6
**Husband /friend occupation**		
Farmer	307	38.2
daily worker	77	9.6
Merchant	219	27.3
government employ	200	24.9
**Total family size**		
1–3	453	56.4
4–6	273	34.0
≥ 7	77	9.6
**Wealth quintile**		
Very poor	42	5.2
Poor	159	19.8
Middle	412	51.3
Better	190	23.7
**Maternal on medication**		
No	49	6.1
Yes	754	93.9

### Maternal, newborn and placental factors

The majority 670 (83.4%) of the mother’s heights were ≥ 150 cm and greater than two-third 592 (73.7%) of their BMI was found > 18.5 kg/m^2^. Greater than half 457 (56.9%) of the newborn’s sex was female, and the majority 696 (86.7%) of the newborn weight was > 2500 g (Table 2).

**Table 2:** Maternal, Newborn and Placental Characteristics in South Gondar Zone Hospitals, Northcentral Ethiopia, 2019

Characteristics	Frequency	Percent
**Known chronic disease**		
No	776	96.6
Yes	27	3.4
**Height of mother**		
< 150 cm	133	16.6
≥ 150 cm	670	83.4
**Sex of the newborn**		
Male	346	43.1
Female	457	56.9
**Maternal BMI**		
< 18.5 kg/m^2^	211	26.3
≥ 18.5 kg/m^2^	592	73.7
**Weight of the newborn**		
< 2500 g	107	13.3
≥ 2500 g	696	86.7
**Placental weight**		
< 350 g	310	38.6
≥ 350 g	493	61.4

### Maternal obstetric factor

Greater than half 461(57.4%) of the mother’s pregnancy interval was < 24 months. Mothers who live within > 3000 m of the altitude above sea level was 443 (55.2%) and attended more than four ANC follow up was 411 (55.92%). The majority 645 (80.3%) of the mothers deliver appropriate gestational age (AGA) babies and their hemoglobin level ≥ 8 g/dl were 798 (99.4%) and had no chronic hypertension were 713(88.8%) (Table 3).

**Table 3:** Obstetric factors of the Mothers in South Gondar Zone Hospitals, Northcentral Ethiopia, 2019

Characteristics	Frequency	Percent
**Pregnancy interval**		
< 24 months	461	57.4
≥ 24 months	325	40.5
Maternal MUAC		
< 23 m	247	30.8
≥ 23 m	556	69.2
**Preterm birth**		
**No**	613	76.84
**Yes**	186	23.16
**Gestational age**		
SGA	158	19.7
AGA	645	80.3
**Altitude**		
< 2000 m	212	26.4
2000–3000 m	148	18.4
> 3000 m	443	55.2
**Gravidity**		
≤ 2	358	44.6
3–4	323	40.2
≥ 5	122	15.2
**Hg after delivery**		
< 8 g/dl	5	0.6
≥ 8 g/dl	798	99.4
**Chronic HPN**		
No	713	88.8
Yes	90	11.2
**PPH**		
No	786	97.9
Yes	17	2.1
**Anemia**		
No	675	84.1
Yes	128	15.9
**Preeclampsia**		
No	700	87.2
Yes	103	12.8
**ANC follow up**		
No	68	8.5
Yes	735	91.5
Total ANC		
< 4 (including no ANC follow up)	411	55.92
≥ 4	324	44.08
**Torch infection**		
No	785	97.8
Yes	18	2.2

### Overall minimum, maximum, and mean of some maternal and newborn characteristics

The minimum age of the respondent in this study was 17 years; whereas the maximum was 46 with a mean ± standard deviation (SD) of 26.9 ± 5.3 years. The minimum and maximum heights of the mothers were 145 and 174 cm respectively, with a mean ± SD of 163 ± 9.3 cm. The minimum weight of the newborn was 1200 g; however, the maximum weight was 4600 g with a mean ± SD of 2768.6 ± 252.4 g (Table 4).

**Table 4:** Minimum, Maximum and Mean + SD value of Maternal Newborn and Placental Parameters in South Gondar Hospitals, Northcentral Ethiopia, 2019

Variable	Minimum	Maximum	Mean **+** SD
Current maternal age	17 years	46 years	26.9 + 5.3 years
Maternal height	145 cm	174 cm	163 + 9.3 cm
Maternal weight	46 kg	82 kg	59 + 10.8 kg
Maternal MUAC	18 cm	36 cm	21 + 2.97 cm
Newborn weight	1200 g	4600 g	2768.6 + 252.4 g
Newborn height	26 cm	56 cm	47.1 + 2.4 centemeters
Newborn head circumference	22 cm	52 cm	35.5 + 4.0 cm
Abdominal circumference	20 cm	48 cm	29.6 + 3.9 cm
Placental weight	320 g	987 g	580 + 270 g
Pregnancy interval	19 months	98 months	34 + 13 months
Gestational age	32 weeks	43 weeks	38 + 3.5 weeks

### Factors associated with intrauterine growth restriction

Since risk factors for IUGR are interrelated, multivariable logistic regression analysis gives more meaningful results: maternal educational status, BMI, MUAC, family size, gestational age, and weight of the placenta were found to be significant predictors of intrauterine growth restriction. Accordingly, the odds of having IUGR were 2 times higher among mothers who were unable to read and write than mothers who achieve their education college and above (AOR; 2.46, 95% CI: 1.02–5.92). The household contains a total family size of ≥7 were 2 times more likely experienced IUGR than household having a total family member of three and less (AOR; 1.67, 95% CI: 1.04–2.66). Mothers MUAC < 23 cm, BMI < 18.5 kg/m^2^ and living in an altitude > 3000 m were 2 times more likely to deliver IUGR newborn compared to mother who had MUAC >23centimetrs (AOR; 2.10, 95% CI: 1.39–3.01), BMI > 18.5 kg/m^2^ (AOR; 2.57, 95% CI: 1.72–3.83) and living at an altitude < 2000 m (AOR; 1.89 95% CI: 1.19–3.01) respectively. Newborn babies exposed to SGA were experienced IUGR than babies had AGA (AOR; 1.94, 95% CI: 1.86–4.52). The odds of developing IUGR from placental weight < 350 g were 2 times as compared with the placental weight > 350 g (AOR; 2.42, 95% CI: 1.67–3.54) ***(***Table 5*).*

**Table 5:** Binary and Multivariable Logistic Regression Analyses of IUGR among Women who Give Birth in South Gondar Zone Hospitals Northcentral Ethiopia, 2019

Variables	IUGR		COR	AOR(95%CI)
	Yes	No		
**Educational status of the mother**				
Unable to read and write	42	180	1.75 (1.03–2.95)	2.46 (1.02–5.92)
Able to read and write	56	217	1.58 (0.96–2.60)	2.39 (1.13–5.03)
Primary	29	52	0.73 (0.40–1.34)	1.01 (0.44–2.30)
Secondary	29	84	1.18 (0.66–2.12)	2.10 (0.94–4.10)
College and above	33	81	1	
**Husband education**				
Unable to read and write	36	150	1.27 (0.75–2.17)	0.67 (0.27_1.62)
Able to read and write	41	174	1.30 (0.77–2.18)	0.78 (0.31–1.68)
Primary	21	45	0.66 (0.34–1.25)	0.71 (0.30–1.68)
Secondary	58	137	0.72 (0.44–1.19)	0.70 (0.36–1.34)
College and above	33	108	1	
**Total family size**				
< 3	126	327	1	
4–6	46	227	1.36 (0.76–2.42)	1.09 (0.50–2.38)
≥ 7	17	60	1.90 (1.30–2.77)	1.67 (1.04–2.66)
**Gravidity**				
≤ 2	90	268	1	
3–4	72	251	1.18 (0.72–1.67)	0.7 (0.39–1.24)
≥ 5	27	95	1.17 (0.82–1.67)	0.64 (0.37–1.13)
**Maternal MUAC**				
< 23 cm	91	156	2.73 (1.94–3.82)	2.10 (1.39–3.01)
≥ 23centimetrs	98	458	1	
**Gestational age**				
SGA	58	79	3.00 (2.03–4.42)	1.94 (1.86–4.52)
AGA	131	535	1	
**Placental weight**				
< 350 g	94	141	3.32 (2.36–4.67)	2.42 (1.67–3.54)
≥ 350 g	95	473	1	
**Altitude**				
< 2000 m	39	173	1	1
2000–3000 m	31	117	1.38 (0.89–2.17)	1.38 (0.81–2.37)
> 3000 m	119	324	1.63 (1.09–2.45)	1.89 (1.19–3.01)
**BMI**				
< 18.5 kg/m^2^	77	134	2.46 (1.74–3.49)	2.57 (1.72–3.83)
≥ 18.5 kg/m^2^	112	480	1	1
**Known chronic disease**				
No	179	597	1	
Yes	10	17	0.51 (0.23–1.13)	0.71 (0.28–1.80)

## Discussions

Intrauterine growth restriction is a commonly faced circumstance in obstetrics, and not only, but it is also associated with perinatal morbidity and mortality. Consequently, it is indispensable to differentiate and diagnose it and take an instantaneous action [26]. As far as our knowledge is concerned, it is the first study in Ethiopia. In this study, therefore, we try to assess the prevalence of IUGR and its associated factors in hospitals of the south Gondar zone, Ethiopia. The data from our study revealed that the prevalence of intrauterine growth restriction was 23.5%, and the rare of low birth weight, small for gestational age, and preterm birth was 13.3, 19.7, and 23.16% respectively. The prevalence of IUGR in this study was similar to other studies that were conducted in Karachi 24.4% [12] and Malawi 20.3% [11]. In Bolivia, a comparison study among people living in high altitude (> 3000 m) and low altitude, newborn babies delivered at high altitude weighted less weight, and also the prevalence of IUGR was 16.8, 95% CI: 14.9–18.6 and 5.9, 95% CI: 4.2–7.5 at higher and lower altitudes respectively [27].

The similarity of these studies might be due to the definition of IUGR and besides, the similarity in Malawi might be due to the study setting which means that both of them conducted in institutions immediately after delivery. However, in this study and Malawi, the studies didn’t include deliveries that took place at home. And our study was higher than the studies held in Brazil at different periods 14.8, 9.4, and 12% [28]. Its difference between this study and in Brazil might be due to the study period.

Generally, maternal educational status was the most significant predictor variables for maternal and child health in the world, similarly, in this study, it was one of the significant variables of IUGR (AOR; 2.46, 95% CI: 1.02–5.92) and it was supported by previous studies conducted in Latin America [29], India [30], and in Karachi (AOR;1.6,95% CI: 1.0–2.7) [12]. This might be because, when a women’s educational level increases, she may be motivated to know health and risk factors, might have the interest to read and listen, watch any information sources, and make an informed decision about their health. Besides, women with some basic level of education can discuss more sensitive issues openly and had a better understanding of the complication associated with pregnancy.

Maternal body mass index is a substantial modifiable risk predictor for intrauterine growth restriction including low birth weight, preterm labor, and small for its gestational age. In this study, maternal Low body mass index (BMI) was a variable associated with intrauterine growth restriction (AOR; 2.57) and it was similar in the study done in Thiruvalla with a *P*-value< 0.001 [31], in developing region [32] and in Karachi (AOR; 2.6, 95% CI: 1.8, 3.7) [12].

High altitude acts as an independently, no interactively with other risk factors to reduce birth weight and mostly pregnancy-associated hypertension was more common at a higher altitude which leads to maternal and neonatal morbidity and mortality [33]. In Bolivia, all maternal, fetal, neonatal complications, including fetal distress (AOR; 7.3 95% CI: 3.9–13.6, hypertensive complications of pregnancy and risk of stillbirth (AOR: 6.0; 95% CI: 2.2–16.2) were more frequent at a higher altitude than lower altitude [27].

As a result, in this study high altitude is a factor of intrauterine growth restriction (AOR; 1.89). This is was in line with a study conducted in Colorado [34] and in Bolivia P-value, 0.001 [27]. IUGR was associated with placental weight in this study, infant borne with low placental weight (< 350 g) were experienced IUGR than placental weight > 350 g (AOR; 2.42). Its contribution is not only for IUGR but also for SGA according to Stanford Medical Center researchers [35] and maternal malnutrition and uteroplacental insufficiency during let pregnancy are usual causes for asymmetric IUGR while congenital infections acquired early in pregnancy have an association with symmetrical IUGR [17]. A comparison study in two cohorts of SGA and AGA showed that the placenta from SGA newborn infants was more likely to have smaller weight and thinner umbilical cords than those from AGA neonates, farther more this smaller placentas had a significant increase in uteroplacental malformation [35]. In this study besides other variables, households total family size ≥7 was a significant variable for IUGR (AOR; 1.67). This might be due to the sharing of foods, inadequate intake due to a higher number of individuals, and the existence of food insecurity that may affect the nutritional status of the members as general, especially the mothers during pregnancy. Because pregnant mothers need additional nutritional supplementation during conception since they are the more vulnerable group for malnutrition. Nutritional intervention could help to increase maternal weight, in particular during pregnancy, and thereby reduce the risk attributable to low maternal weight.

## Conclusions

In our knowledge, even though this study was the first study in the country, the prevalence of intrauterine growth restriction is a major Public Health concern. Maternal education, gestational age, BMI, family size in the household, maternal mid-upper arm circumference, placental weight, and altitude were found the most predictor variable. To avert intrauterine growth retardation, decreasing small for gestational age and increasing placental weight is very essential through additional nutritional supplementation during pregnancy. Because, when maternal weight increased, IUGR/ SGA will be substantially decreased.

Education is the core center of knowledge, so the government should address at least primary education for females. Health professionals including health extension workers shall have to counsel women about the importance of birth interval (interpregnancy interval), nutrition, and giving health care for women before (preconception care) and during pregnancy (antenatal care follow up) and nutritional intervention could help to increase maternal weight and thereby reduce the risk attributable to IUGR. The health care providers should give special attention to pregnant women living in high altitudes, critically to their weight (following pregnancy weight gain in each trimester). Obstetricians and perinatologist need to recognize the fetus(es) at risk of IUGR, to identify the modifiable risk factors and optimize the maternal systemic diseases. Further clinical follow-up research is essential which includes placental, maternal, and fetal gene.

## Data Availability

All the data sets are available on the hand of the corresponding author.

## Abbreviations

BMI: Body Mass Index
BP: Blood pressure
Cm: Centimeter
EDHS: Ethiopian demographic and health survey
GH: Gestational Hypertension
Kg: Kilogram
M: Meter
IUGR: Intrauterine growth restriction/restriction
IUM: Intrauterine mortality
MUAC: Mid-Upper Arm Circumference
PE: Preeclampsia
OR: Odds Ratio
SGA: Small-for-Gestational Age
